# Semi-Synthesis, Anti-Leukemia Activity, and Docking Study of Derivatives from 3*α*,24-Dihydroxylup-20(29)-en-28-Oic Acid

**DOI:** 10.3390/molecules30153193

**Published:** 2025-07-30

**Authors:** Mario J. Noh-Burgos, Sergio García-Sánchez, Fernando J. Tun-Rosado, Antonieta Chávez-González, Sergio R. Peraza-Sánchez, Rosa E. Moo-Puc

**Affiliations:** 1 Unidad de Biotecnología, Centro de Investigación Científica de Yucatán (CICY), Mérida 97205, Mexico; marionohburgos17@gmail.com (M.J.N.-B.);; 2 Unidad Médica de Alta Especialidad, Centro Médico Ignacio García Téllez, Instituto Mexicano del Seguro Social (IMSS), Mérida 97200, Mexico; 3 Unidad de Investigación Médica en Enfermedades Oncológicas, UMAE Hospital de Oncología, Centro Médico Nacional Siglo XXI, IMSS, Ciudad de México 06720, Mexico; 4 Instituto de Química, Universidad Nacional Autónoma de México, Ciudad de México 04510, Mexico; 5 Secretaría de Ciencia, Humanidades, Tecnología e Innovación-Hospital Regional de Alta Especialidad de la Península de Yucatán, IMSS-BIENESTAR, Mérida 97130, Mexico

**Keywords:** leukemia, pentacyclic lupane-type triterpenes, apoptosis, docking studies

## Abstract

Current treatments against leukemia present several limitations, prompting the search for new therapeutic agents, particularly those derived from natural products. In this context, structural modifications were performed on the triterpene 3*α*,24-dihydroxylup-20(29)-en-28-oic acid (**T1**), isolated from *Phoradendron wattii*. Among the five derivatives obtained, 3*α*,24-dihydroxy-30-oxolup-20(29)-en-28-oic acid (**T1c**) exhibited the highest activity, with an IC_50_ value of 12.90 ± 0.1 µM against THP-1 cells. **T1c** significantly reduced cell viability in both acute lymphoblastic leukemia (CCRF-CEM, REH, JURKAT, and MOLT-4) and acute myeloid leukemia (THP-1) cell lines, inducing apoptosis after 48 h of treatment, while showing minimal cytotoxicity toward normal mononuclear cells (MNCs). In silico molecular docking studies were conducted against three key protein targets: BCL-2 (B-cell lymphoma 2), EGFR (epidermal growth factor receptor, tyrosine kinase domain), and FLT3 (FMS-like tyrosine kinase 3). The lowest binding energies (kcal/mol) observed were as follows: **T1**–BCL-2: −10.12, EGFR: −12.75, FLT3: −14.05; **T1c**–BCL-2: −10.23, EGFR: −14.50, FLT3: −14.07; **T2**–BCL-2: −11.59, EGFR: −15.00, FLT3: −14.03. These findings highlight **T1c** as a promising candidate in the search for anti-leukemic drugs which deserves further study.

## 1. Introduction

Globally, cancer is the second leading cause of death, with 20 million new cases and 9.7 million deaths reported in 2022 [1,2]. Leukemias rank thirteenth among the most common cancers and represent the tenth leading cause of cancer-related death worldwide [2]. Among the types of acute leukemia, acute lymphoblastic leukemia (ALL) is most frequently diagnosed in children and young adults, while acute myeloid leukemia (AML) is more prevalent in adults but is also common in children [3,4,5].

The treatment of choice for leukemia is allogeneic hematopoietic stem cell transplantation [6]. However, it presents major challenges such as the lack of compatible donors and its low efficacy in advanced stages of the disease [6]. Chemotherapy continues to be widely used but faces common challenges in oncology, such as toxicity, adverse side effects, development of cell drug resistance mechanisms, and disease recurrence [7,8,9]. Therefore, the search for new, more effective, and selective drugs against leukemic cells remains an ongoing challenge [8,9].

Natural products have been widely used in the discovery and development of new drugs. It is estimated that 40% of the drugs used in cancer treatment have been inspired by natural products. The isolation of bioactive compounds from different natural sources, together with the generation of structural analogues of these compounds by chemical derivatization, are the most important strategies in the search for anticancer drugs [10,11].

Pentacyclic lupane-type triterpenes (PLTTs) are compounds isolated from natural sources that exhibit notable chemopreventive and antineoplastic properties, but face limitations such as low solubility, bioavailability, and selectivity [12,13,14,15]. To address these challenges, researchers have synthesized derivatives and employed strategies like complexation with cyclodextrins, liposomes, carbon nanotubes, or gold nanoparticles [16,17,18]. Additionally, introducing delocalized lipophilic cationic groups (e.g., TPP, F16, and Rho123) has enhanced their stability, solubility, and selective targeting of tumor cells, improving their therapeutic potential in cancer treatment [19,20].

PLTTs serve as a valuable scaffold for developing compounds with enhanced anti-leukemic activity due to their modifiable positions (C-3, C-17, C-20, and C-28), which improve activity and selectivity against cancer cells [15,21]. For instance, introducing a 1,2,3-triazole at C-3 of betulinic acid enhances activity in acute myeloid leukemia (HL-60 and THP-1) cells [15]. Similarly, adding a propynoyl group at C-28 of betulin increases cytotoxicity in acute lymphoblastic leukemia (CCRF-CEM) and murine leukemia (P388) cells [22]. Additionally, modifying betulin at C-3 with a 4-fluorophenylhydrazone group significantly improves activity against MOLT-4 leukemia cells [23].

Recently, studies have highlighted the importance of structural modifications at the C-30 position of triterpenes to improve their bioactivity and reduce their cytotoxicity in normal cells [7]. For example, the introduction of an aldehyde group on C-30 of betulinic acid resulted in the formation of 30-oxobetulinic acid with cytotoxic activity on cancer and CCRF-CEM leukemia cell lines [24]. In this context, the presence of the formyl group, in the form of *α*,*β*-unsaturated aldehyde on C-30, contributed to obtaining compounds more active against leukemic cells compared to their non-functionalized analogues [25,26,27].

**Figure 1:**
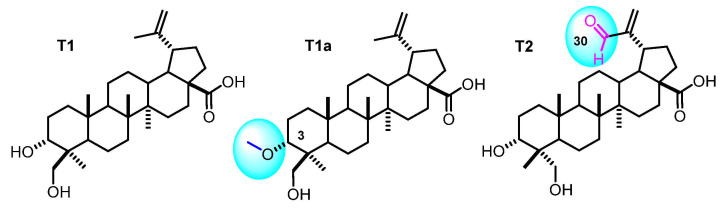
Molecular structures of **T1**, **T1a**, and **T2**.

Previous studies evaluated the pentacyclic triterpenes 3*α*,24-dihydroxylup-20(29)-en-28-oic acid (**T1**) and 3*α*,23-dihydroxy-30-oxolup-20(29)-en-28-oic acid (**T2**) (Figure 1), isolated from *Phoradendron wattii* [28], in chronic (K562) and acute (HL60) myeloid leukemia cell lines. **T2** induced cell cycle arrest at G2/M, likely due to its *α*,*β*-unsaturated aldehyde at C-30, which enhanced activity against leukemic cells compared to **T1** [29]. Additionally, modifying **T1** by introducing a methyl group at C-3 (3*α*-methoxy-24-hydroxylup-20(29)-en-28-oic acid, **T1a**) improved its anti-leukemic effects, including apoptosis induction, ROS generation, and mitochondrial membrane disruption [30].

In this context, PLTTs, specifically those isolated from *P. wattii*, present favorable characteristics such as good yields, structural stability, biological activity, and selectivity [28,29,30]. The triterpene **T1** represents an important scaffold due to the presence of promising sites, such as C-3 and C-30, making it a good candidate for simple structural modifications with the intention of generating new semi-synthetic derivatives with improved biological activity on acute lymphoblastic leukemia cell lines (CCRF-CEM, REH, JURKAT, and MOLT-4) and an acute myeloid leukemia cell line (THP-1).

## 2. Results and Discussion

### 2.1. Chemical Synthesis of the Target Compounds

Due to the cytotoxic effects observed for compound **T1**, its derivative **T1a**, and compound **T2** in leukemia cell lines [29,30], in the present study, a series of **T1** analogues were developed by minor structural modifications to be evaluated in a panel of leukemia cell lines.

**Scheme 1:**
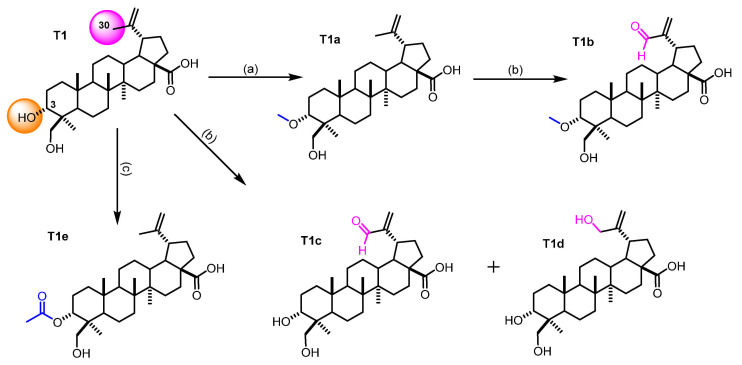
Semi-synthesis of 3*α*,24-dihydroxylup-20(29)-en-28-oic acid derivatives (**T1a**–**T1e**). Reagents and conditions: (a) CH_3_I, K_2_CO_3_, Me_2_CO anh., room temperature, 24 h; (b) SeO_2_, EtOH anh., reflux 48 h; (c) AcO_2_, pyridine, room temperature, 72 h.

Compound **T1** was isolated from the aerial parts of *P. wattii* according to a previous study [28]. Derivatives were obtained from **T1** by a series of reactions targeting the C-3 and C-30 carbons. Initially, **T1** was subjected to a C-3 methylation reaction employing CH_3_I/K_2_CO_3_ in anhydrous Me_2_CO, resulting in the derivative 3*α*-methoxy-24-hydroxylup-20(29)-en-28-oic acid (**T1a**) with a 68.4% yield (Scheme 1, step a). The structure of **T1a** was confirmed based on the ^1^H-NMR spectrum (Figure S1, Supplementary Materials); a new single signal at *δ* 3.66 characteristic of a methyl group attached to a heteroatom was observed. Furthermore, the ^13^C-NMR spectrum (Figure S2) showed a new signal at *δ* 51.4. These results confirmed that **T1a** is 3*α*-methoxy-24-hydroxylup-20(29)-en-28-oic acid. Subsequently, this compound (**T1a**) reacted with SeO_2_ in anhydrous EtOH, carrying out an allylic oxidation reaction on C-30, yielding the acid derivative 3*α*-methoxy-24-hydroxy-30-oxolup-20(29)-en-28-oic acid (**T1b**) with a 50% yield (Scheme 1, step b). In the ^1^H-NMR spectrum (Figure S3), the absence of the signal at *δ* 1.60–1.80, corresponding to the H-30 protons, is noticeable, and a new signal at *δ* 9.52, characteristic of aldehyde groups, can be observed. Furthermore, in the ^13^C-NMR spectrum (Figure S4) the signal at *δ* 194.1 was assigned to this aldehyde group at C-30. These results confirm that **T1b** is 3*α*-methoxy-24-hydroxy-30-oxolup-20(29)-en-28-oic acid.

On the other hand, under the same conditions as for **T1b**, an allylic oxidation reaction was carried out at the C-30 position of compound **T1**, yielding the acid derivative 3*α*,24-dihydroxy-30-oxolup-20(29)-en-28-oic acid (**T1c**) with a 17% yield and the by-product 3*α*,24,30-trihydroxylup-20(29)-en-28-oic acid (**T1d**) with a 19% yield. In the ^1^H- and ^13^C-NMR spectra for **T1c** (Figures S5 and S6), similar signals were observed as for **T1b**, a new signal at *δ* 9.51 characteristic of aldehyde groups and a signal at *δ* 196.9, respectively. On the other hand, the structure of the by-product **T1d** was confirmed based on the ^1^H-NMR spectrum (Figure S7); a new signal was observed at *δ* 4.05 (*J*_AB_ = 15.8 Hz, 2H) with three lines, the inner signal overlapped, and the exterior signals were very attenuated (roof effect). This pattern is characteristic of AB systems and corresponds to the isolated geminal nuclei in H-30 adjacent to the hydroxyl group. Furthermore, in the ^13^C-NMR spectrum (Figure S8), a new signal was observed at *δ* 65.2 that corresponds to the oxygen-bound methylene of C-30. These results confirm that **T1d** is 3*α*,24,30-trihydroxylup-20(29)-en-28-oic acid.

Compound **T1d** was obtained through a selective oxidation of the allylic system present in the precursor triterpene, using selenium dioxide (SeO_2_) as the oxidizing agent. This type of transformation is a well-known allylic oxidation that proceeds via a mechanism involving an Alder-ene-type cycloaddition, followed by a [2,3]-sigmatropic rearrangement, which generates an unstable seleninic intermediate. Hydrolysis of this intermediate and elimination of reduced selenium species (Se(IV)) lead to the formation of the allylic alcohol at the C-30 position, corresponding to compound **T1d** [31].

Finally, C-3 acetylation of **T1** employing acetic anhydride yielded the derivative 3*α*-acetyl-24-hydroxylup-20(29)-en-28-oic acid (**T1e**) with a 62% yield (Scheme 1, step c). The structure of **T1e** was confirmed based on the ^1^H-NMR spectrum (Figure S9), a new single signal at *δ* 2.02 ppm, corresponding to the methyl group of the acetyl substituent. In addition, in the ^13^C-NMR spectrum, two new signals were observed at *δ* 171.5 and *δ* 21.4 ppm corresponding to the carbonyl and methyl groups of the acetyl substituent, respectively. Under mild acetylation conditions (Ac_2_O/pyridine, room temperature), only the C-3 hydroxyl group was acetylated, while the C-24 hydroxyl remained unchanged. This selectivity can be explained by the greater accessibility and nucleophilicity of the equatorial C-3 hydroxyl, whereas the C-24 hydroxyl is less accessible due to steric hindrance from the rigid lupane side chain. Consistently, NOESY data from lupane-type triterpenes isolated from *P. wattii* showed that H-24 and H-25 are spatially close, indicating that the C-24 hydroxyl resides in a hindered environment, which likely limits its reactivity [28].

### 2.2. In Vitro Anti-Leukemia Activity

The viability effect of **T1**, **T2**, and **T1** derivatives using different concentrations was evaluated on five leukemia cell lines: CCRF-CEM, REH, JURKAT, MOLT-4, and THP-1, the results being compared with dasatinib (Table 1). Of particular interest, the derivatives **T1b** and **T1c** showed significant activity in all cell lines evaluated, with IC_50_ values lower than that of the parent compound **T1**, which showed no activity, while **T2** showed greater selectivity against the CCRF-CEM and REH cell lines compared to that against the JURKAT cell line. On the other hand, the **T1a** derivative showed moderate activity on the REH cell line (IC_50_ = 64.22 µM), while **T1d** and **T1e** showed no activity.

Among all the derivatives, compounds **T1b** and **T1c** exhibited the highest activity across the tested cell lines. Notably, both contain an *α*,*β*-unsaturated aldehyde group at C-30, a key structural feature associated with increased cytotoxicity. The methylation of the hydroxyl group at C-3 in **T1b** further enhanced its activity. *α*,*β*-Unsaturated carbonyl groups, such as aldehydes and ketones, exert cytotoxic effects by forming Michael-like adducts, as the electrophilic *β*-carbon readily reacts with thiol groups in proteins and peptides involved in cancer progression and drug resistance. Beyond their interaction with cellular thiols, these groups can also trigger apoptosis via the mitochondrial pathway, a crucial mechanism of cell death and a promising target for cancer therapy [32,33].

For example, oxidation at C-30 of lupeol, which leads to the formation of an *α*,*β*-unsaturated aldehyde in the isopropylidene fragment, has produced derivatives with cytotoxic activity against leukemia cell lines. The compound 3*β*-hydroxylup-20(29)-en-30-al showed IC_50_ values of 11.72 ± 1.06 µM in JURKAT cells and 19.52 ± 0.47 µM in K562 cells [7]. However, this compound also reduced the viability of normal peripheral blood lymphocytes (PBLs) by 32.17% compared to untreated controls, suggesting a potential cytotoxic effect on non-tumor cells [7]. In a previous study, another 30-oxo-lupane compound (**T2**) was reported to reduce K562 cell viability to 88.6 ± 0.40% at 102 µM and to 24.3 ± 1.95% at 204 µM, while showing a moderate effect on HL-60 cells (88.35 ± 3.85% and 85.15 ± 3.05%, respectively) [29].

In comparison, compounds **T1b** and **T1c** exhibited potent cytotoxic activity across all leukemia cell lines tested, with IC_50_ values ranging from 4.05 to 14.97 µM for **T1b** and from 12.90 to 24.92 µM for **T1c**. These results highlight the significance of introducing an *α*,*β*-unsaturated carbonyl group at C-30 as a key structural modification to enhance cytotoxic potential in leukemia models, while also emphasizing the importance of assessing selectivity toward cancer cells. In this context, previous studies have demonstrated that incorporating a formyl group at C-30 is a critical modification, yielding derivatives with markedly higher activity against leukemic cells compared to the precursor molecule [7,24,32].

On the other hand, compounds **T1c** and **T2** are epimers, differing in the orientation of the hydroxymethyl group at the C-4 position, either forward (C-24) or backward (C-23), respectively. Compound **T1c** showed a significant effect on all cell lines tested, whereas **T2** generated less effect on CCRF-CEM, REH, and JURKAT cell lines. Based on these differences in activities and stereochemistry, interaction with enzymes or receptors is possibly the main mechanism of action involved [29].

### 2.3. Effect of Compounds on the Viability of Peripheral Blood Mononuclear Cells (MNCs)

The safety of the compounds was measured by assessing the viability effect on MNCs (Table 2). When the **T1b** derivative was evaluated at the maximum concentration of 200 µM, it showed a potent cytotoxic effect on normal cells (IC_50_ = 10.78 µM), while **T1c** showed a non-significant effect on MNCs, implying a high selectivity (SI > 10) of this derivative against leukemia cell lines. On the other hand, **T1a**, **T1d**, and **T1e** showed no activity at the maximum concentration evaluated.

The **T1b** derivative was cytotoxic in normal MNC cells. Therefore, simple modifications such as the introduction of methyl groups together with the presence of the formyl group in this type of triterpenes may confer cytotoxic effects on normal MNC cells. The presence of the Michael-type acceptor on C-30 could facilitate their covalent binding to proteins, which contributes to their cytotoxicity [33]. Therefore, structural modifications can be made by replacing the formyl group with hydrazones or azines, which can interact in a similar manner [24]. One study showed that the introduction of asymmetric azines into 30-oxobetulinic acid generated derivatives selective for CCRF-CEM leukemia cells and proved to be inactive in non-malignant human fibroblasts [24]. Therefore, the design and structural modification of **T1b** could be oriented towards the introduction of azine groups to improve its selectivity.

### 2.4. Apoptosis Assay

Since compound **T1c** was the only derivative that decreased the cell viability of leukemic cells (IC_50_ ≤ 20 µΜ), without affecting viability in normal MNC cells, its mode of action to exert cytotoxic effects was evaluated by flow cytometry techniques, determining the apoptotic effect at 48 h, using ½ IC_50_ and IC_50_ concentrations obtained in the viability assay in the different leukemia cell lines. Dasatinib at different concentrations was included as a positive control. Cell death was quantified by dual staining with annexin V-FITC and DAPI. Annexin V binds to externalized phosphatidylserine residues during early and late apoptosis, whereas DAPI penetrates membrane-compromised cells, distinguishing between late apoptotic and necrotic cells [34,35]. The total percentage of apoptotic cells was calculated as the sum of early and late apoptotic populations. The results were normalized to 100% with respect to the negative control.

To better understand the type of cell death induced by **T1c**, it is essential to distinguish the mechanistic differences between early and late apoptotic processes. Early apoptosis involves the activation of initiator caspases (caspase-8 or -9) and mitochondrial outer membrane permeabilization (MOMP), mediated by Bax/Bak oligomerization. This leads to cytochrome c release and apoptosome formation. At this stage, phosphatidylserine is externalized to the outer leaflet of the plasma membrane, without loss of membrane integrity. In contrast, late apoptosis is characterized by the activation of executioner caspases (caspase-3, -6 and -7), which degrade structural proteins and activate caspase-activated DNase (CAD), resulting in nuclear condensation, internucleosomal DNA fragmentation, formation of apoptotic bodies, and irreversible cell disintegration. These events ensure the efficient and controlled elimination of apoptotic cells, minimizing the risk of triggering inflammatory responses [36,37,38,39].

**Figure 2:**
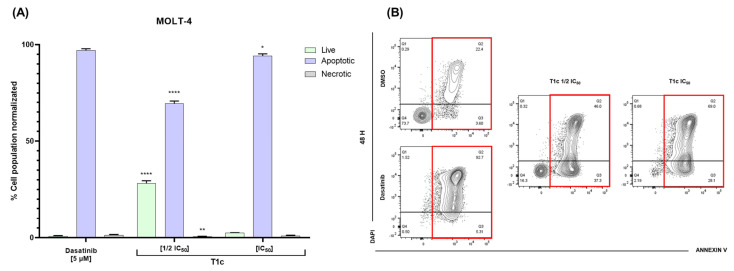
(**A**) **T1c** induced apoptosis in the MOLT-4 cell line. **T1c** was evaluated at ½ IC_50_ and IC_50_ exposed for 48 h. * *p* < 0.05, ** *p* < 0.01, **** *p* < 0.0001 were compared with the positive control. Statistical significance was calculated using one-way analysis of variance (ANOVA) followed by Dunnett’s post hoc analysis. (**B**) Representative dot plots of MOLT-4 cultures, treated with vehicle control (DMSO), positive control (dasatinib), and **T1c** for 48 h. Cluster Q1: Necrotic or other death processes; Cluster Q2: Late apoptotic cells; Q3: Early apoptotic cells; Q4: Living cells. Apoptotic cell population in red box.

In the present results, it was observed that **T1c** induces apoptotic death in leukemic cells to varying degrees. For example, for the MOLT-4 cell line (Figure 2), the apoptosis rate was 96.13 ± 0.71% (early apoptotic cells: 1.40%; late apoptotic cells: 94.74%) for the positive control, while **T1c** at ½ IC_50_ exhibited apoptosis rates of 69.91 ± 2.17% (early apoptotic cells: 41.15%; late apoptotic cells: 28.76%). When the IC_50_ was evaluated, the apoptosis rate changed to 94.58 ± 0.45% (early apoptotic cells: 30.12%; late apoptotic cells: 64.46%), similar to the positive control. These results highlight the apoptotic effect of **T1c** on the MOLT-4 cell line.

**Figure 3:**
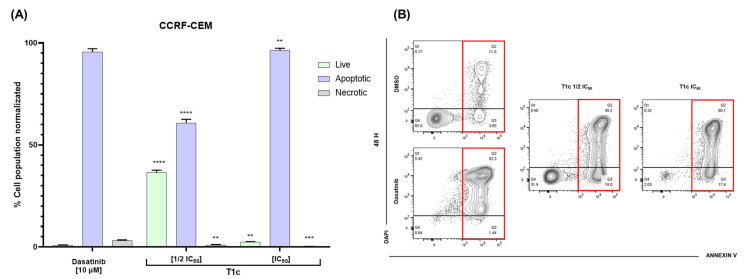
(**A**) **T1c** induced apoptosis in the CCRF-CEM cell line. **T1c** was evaluated at ½ IC_50_ and IC_50_ exposed for 48 h. ** *p* < 0.01, *** *p* < 0.001, **** *p* < 0.0001 were compared with the positive control. Statistical significance was calculated using one-way analysis of variance (ANOVA) followed by Dunnett’s post hoc analysis. (**B**) Representative dot plots of CCRF-CEM cultures, treated with vehicle control (DMSO), positive control (dasatinib), and **T1c** for 48 h. Cluster Q1: Necrotic or other death processes; Cluster Q2: Late apoptotic cells; Q3: Early apoptotic cells; Q4: Living cells. Apoptotic cell population in red box.

Under the same conditions, for the CCRF-CEM cell line at 48 h of treatment, the apoptosis rate was 94.84 ± 1.07% (early apoptotic cells: 1.66%; late apoptotic cells: 92.90%) in the positive control (Figure 3), while for **T1c** at ½ IC_50_ concentration it was 62.40 ± 1.57% (early apoptotic cells: 16.98%; late apoptotic cells 45.42%). However, when evaluated at the IC_50_ concentration, an increase in apoptosis of 97.13 ± 0.36% (early apoptotic cells: 16.92%; late apoptotic cells: 80.21%) similar to the positive control was observed.

**Figure 4:**
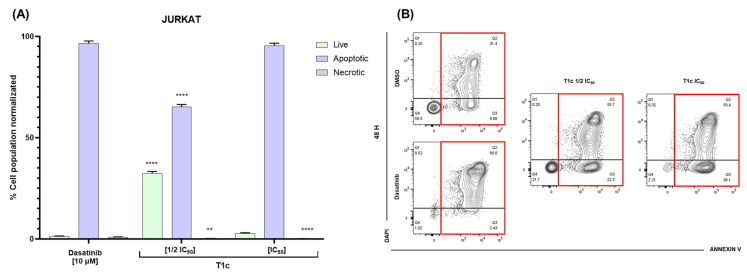
(**A**) **T1c** induced apoptosis in the JURKAT cell line. **T1c** was evaluated at ½ IC_50_ and IC_50_ exposed for 48 h. ** *p* < 0.01, **** *p* < 0.0001 were compared with the positive control. Statistical significance was calculated using one-way analysis of variance (ANOVA) followed by Dunnett’s post hoc analysis. (**B**) Representative dot plots of JURKAT cultures, treated with vehicle control (DMSO), positive control (dasatinib), and **T1c** for 48 h. Cluster Q1: Necrotic or other death processes; Cluster Q2: Late apoptotic cells; Q3: Early apoptotic cells; Q4: Living cells. Apoptotic cell population in red box.

On the other hand, when assayed against the JURKAT cell line (Figure 4), the positive control showed 97.19 ± 0.11% apoptosis (early apoptotic cells: 0.59%; late apoptotic cells: 96.60%). The **T1c** derivative showed apoptosis rates of 67.14 ± 2.18% (early apoptotic cells: 30.60%; late apoptotic cells: 36.55%) at the concentration of ½ IC_50_. When evaluated at IC_50_, the apoptosis rate changed to 96.37 ± 0.49% (early apoptotic cells: 47.61%; late apoptotic cells: 48.72), similar to that of the positive control.

**Figure 5:**
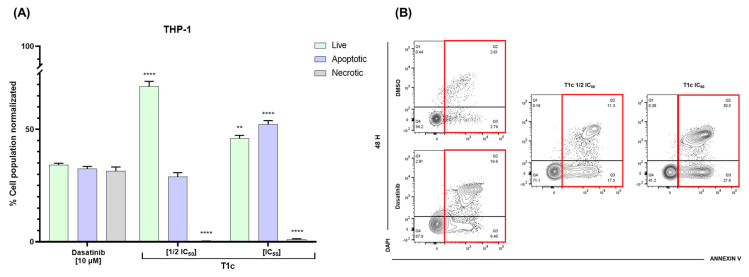
(**A**) **T1c** was evaluated at ½ IC_50_ and IC_50_ exposed for 48 h in the THP-1 cell line. ** *p* < 0.01, **** *p* < 0.0001 were compared with the positive control. Statistical significance was calculated using one-way analysis of variance (ANOVA) followed by Dunnett’s post hoc analysis. (**B**) Representative dot plots of THP-1 cultures, treated with vehicle control (DMSO), positive control (dasatinib), and **T1c** for 48 h. Cluster Q1: Necrotic or other death processes; Cluster Q2: Late apoptotic cells; Q3: Early apoptotic cells; Q4: Living cells. Apoptotic cell population in red box.

For the acute myeloid leukemia cell line THP-1 (Figure 5), the positive control showed 32.14 ± 3.15% apoptosis (early apoptotic cells: 7.21%; late apoptotic cells: 24.93%). The **T1c** derivative showed an effect at ½ IC_50_ of 29.40 ± 2.66% (early apoptotic cells: 14.50%; late apoptotic cells: 14.90%). When IC_50_ values were used, death by apoptosis was clearly higher at 52.23 ± 1.69% (early apoptotic cells: 22.84%; late apoptotic cells: 29.39%) than the positive control. Thus, the THP-1 line is clearly less sensitive to this compound than the rest of the cell lines. The **T1c** derivative induces apoptotic death in leukemic cells to varying degrees.

### 2.5. Molecular Docking

The ability of the **T1c** derivative to induce apoptosis in acute lymphoblastic leukemia (ALL) and acute myeloid leukemia (AML) cell lines highlighted the importance of performing molecular docking analysis with key targets in the search for new compounds with anti-leukemic activity. For this purpose, the Molecular Operating Environment (MOE) software was used [40], focusing on key leukemia-associated targets: the first one was BCL-2, which is related to the control and regulation of apoptosis; this protein blocks apoptosis through the intrinsic pathway [38,39,41]. The second one was the epidermal vascular endothelial growth factor receptor (EGFR), which plays a key role in cell survival and proliferation; in addition, it has downstream signaling pathways associated with the BCL-2 family [42,43,44]. Finally, the third one was the FMS-like tyrosine kinase 3 (FLT3) gene, which is mainly expressed in the bone marrow, especially in CD34+ hematopoietic stem cells and early progenitor cells of the myeloid and lymphoid lineages; besides, it has downstream signaling pathways associated with PI3K/AKT, RAS/MAPK, and STAT5 that promote proliferation and suppress apoptosis [45,46,47]. The docking scores of compounds **T1**, **T2**, and **T1c** were compared with betulinic acid and a co-crystallized inhibitor (Table 3).

#### 2.5.1. Molecular Docking with the BCL-2 Protein

**Figure 6:**
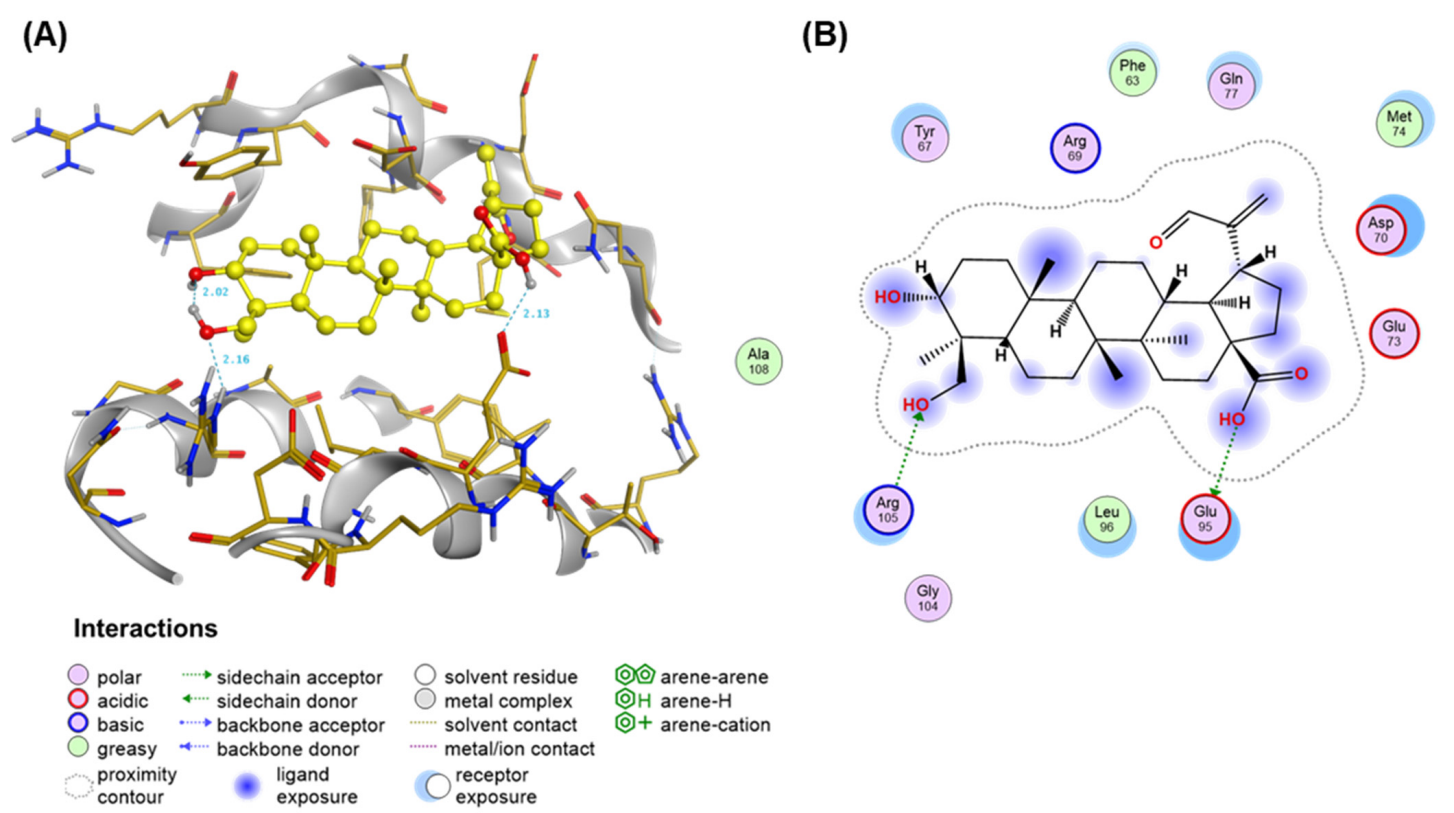
Binding modes of **T1c** with BCL-2 protein. (**A**) Compound **T1c** docked to BCL-2 binding site. (**B**) Two-dimensional interactions between **T1c** and BCL-2 protein.

Molecular docking analysis with the BCL-2 protein revealed that the **T1c** derivative presented an affinity energy similar to that of its molecular precursor (**T1**) and a lower affinity than its **T2** epimer. Relative to betulinic acid, **T1c** showed better affinity (−10.23 kcal/mol). However, the co-crystallized inhibitor presented the best affinity score (−17.72 kcal/mol). Taken together, these results indicate that the effects observed for compounds **T1c** and **T2** are not directly related to BCL-2 inhibition. The crystal structures of BCL-2 in complex with a BH3 domain inhibitor were used. Figure 6 shows the different binding modes of the most active compound (**T1c**) with this key molecular target.

#### 2.5.2. Molecular Docking with the EGFR Tyrosine Kinase

**Figure 7:**
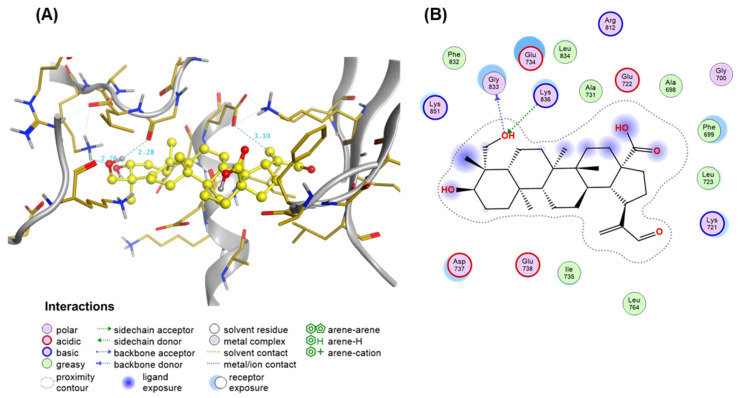
Binding modes of **T1c** with EGFR tyrosine kinase. (**A**) **T1c** docked to EGFRTyrKin binding site. (**B**) Two-dimensional interactions between **T1c** and **EGFRTyrKin**.

The crystal structures of EGFR with erlotinib inhibitors were used. In the present docking results with the EGFR kinase domain, the **T1c** derivative (−14.50 kcal/mol) showed higher affinity than its precursor molecule, **T1** (−12.75 kcal/mol), betulinic acid (−12.73 kcal/mol), and even presented better affinity energy than the co-crystallized inhibitor (−13.97 kcal/mol), and, furthermore, it presented similar affinity energy to its epimer compound, **T2** (−15.00 kcal/mol). On the other hand, the **T1c** derivative showed two favorable interactions at the binding site through hydrogen bridge interactions with residues GLY833 and LYS836 (Figure 7). These results suggest that the apoptotic effect observed during the experimental analysis could be due to the interaction with the EGFR molecular target, which in turn can trigger cell death in the different cell lines evaluated.

#### 2.5.3. Molecular Docking with FLT3 Protein

**Figure 8:**
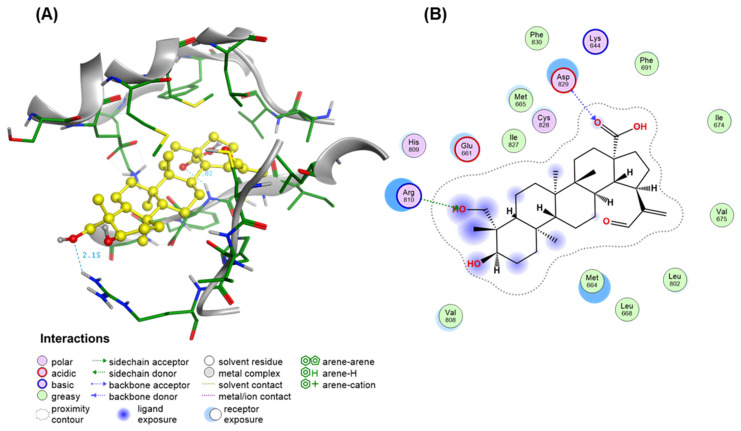
Binding modes of **T1c** with FLT3 protein. (**A**) Compound **T1c** docked to FTL-3 binding site. (**B**) Two-dimensional interactions between **T1c** and **FLT-3**.

Finally, for the FLT3 kinase domain, the binding energies of compounds **T1**, **T2**, and the **T1c** derivative were found to be slightly more affine than betulinic acid (−13.64 kcal/mol) and showed a lower affinity than the co-crystallized inhibitor (−18.17 kcal/mol). Based on the docking scores for interaction with FLT-3, none of the exerted effects of the **T1c** derivative were found to be related to this target. The crystal structures of FLT3 with quizartinib inhibitors were used. Figure 8 shows the different binding modes of **T1c** with this key molecular target.

#### 2.5.4. Similarity to Drugs

As a complementary study, we submitted the molecular structures of the four compounds to in silico analysis via the SwissADME platform to predict their physicochemical and pharmacokinetic properties (Table 3).

Lipinski’s rule of six was applied for betulinic acid, **T1**, **T2**, and the **T1c** derivative. Of all the compounds, the **T1c** derivative and its epimer compound **T2** complied with all the rules, with acceptable values for molecular weight (≤500), hydrogen bond donors (nHBA ≤ 5), hydrogen bond acceptors (nHBD ≤ 10), lipophilicity (expressed as cLogP ≤ 5), molar refractivity (TPSA ≤ 140 Å), and rotatable bonds (nROTB ≤ 10) [48,49]. On the other hand, results from the SwissADME platform showed that the **T1c** derivative and **T2** have a higher probability of passive absorption in the gastrointestinal tract than **T1** and betulinic acid. In addition, **T1c** and **T2** are predicted not to inhibit with some of the key CYP450 isoforms (Table 4) [48]. The results obtained suggest the **T1c** derivative as a good candidate for further studies.

The results suggest that the **T1c** derivative is a good candidate for future studies on its role in the treatment of leukemia, as it is able to induce apoptosis in ALL and AML cells, as well as potentially act as an EGFR inhibitor. Notably, the SwissADME platform showed promising results for the **T1c** derivative, as it complies with all Lipinski’s rules, with good water solubility (Log P: 4.66), and it has a higher probability of passive absorption in the gastrointestinal tract. In addition, **T1c** is predicted to be a non-substrate of P-glycoprotein, which is a positive feature, since this drug efflux pump has been linked to anticancer drug resistance due to low drug accumulation in multidrug-resistant cells. Putting all this together, it can be stated that **T1c** is a promising molecule. Therefore, gaining a thorough understanding of its mode of action, exploring gene and protein expression, evaluating the effects of **T1c** on leukemia stem cells, and conducting in vivo bioavailability and toxicity studies are crucial.

## 3. Materials and Methods

### 3.1. General Experimental Procedures

NMR spectra were recorded in CDCl_3_ and CD_3_OD on a Bruker Avance 400 spectrometer (Billerica, MA, USA). All chemical shifts (*δ*) were referenced to the residual signals of the deuterated solvent, and coupling constants are reported in hertz (Hz).

Thin-layer chromatography (TLC) was performed on an aluminum sheet precoated with silica gel 60 F_254_ (Sigma-Aldrich, Saint Louis, MO, USA), using a layer of 0.25 mm for analytical purposes and 0.5 mm for preparative application. Spots were detected under UV light at 254 and 366 nm or after spraying with a mixture of H_2_SO_4_-AcOH-H_2_O (1:20:4). Column chromatography (CC) was carried out, employing silica gel 60 with particle sizes ranging from 63 to 200, 40 to 63, or 2 to 25 µm, as well as Sephadex LH-20 (Sigma-Aldrich), depending on the purification needs.

All bioassays were conducted under sterile conditions inside a Class II, Type A2 laminar flow hood (NuAire, Plymouth, MN, USA). Cell cultures were maintained in a NuAire CO_2_ incubator equipped with a water-jacketed chamber and an HEPA filtration system.

### 3.2. Extraction and Isolation of Compounds

In a previous study, the compounds 3*α*,24-dihydroxylup-20(29)-en-28-oic acid (**T1**) and 3*α*,23-dihydroxy-30-oxolup-20(29)-en-28-oic acid (**T2**) were isolated from the aerial parts of *Phoradendron wattii* [28]. Briefly, the dried aerial material of *P. wattii* was macerated at room temperature with methanol. The methanolic extract obtained was concentrated under reduced pressure and suspended in a hydroalcoholic solution of methanol and water (1:3). Subsequently, the mixture was successively partitioned with hexane, dichloromethane, and ethyl acetate to obtain the different organic fractions. The hexane and dichloromethane fractions were purified by silica gel column chromatography, using different solvent systems, to isolate compounds **T1** and **T2**. Structural elucidation was performed by NMR spectroscopy as well as high-resolution mass spectrometry (HRMS) [28].

### 3.3. Synthesis of Compound T1a from T1

A mixture of **T1** (100 mg, 0.21 mmol) and K_2_CO_3_ (540 mg, 3.90 mmol) was solubilized in 2.5 mL of CH_3_I and 5 mL of anhydrous acetone. The reaction was stirred at room temperature for 24 h. After completion, the mixture was subjected to liquid–liquid extraction with H_2_O and EtOAc (1:2 × 2, followed by 1:1). The combined organic layers were dried over Na_2_SO_4_, and the solvent was removed under reduced pressure [30]. The crude product was purified by column chromatography (hexane-acetone, 80:20) to yield 78 mg (75.7%) of 3*α*-metoxy-24-hydroxylup-20(29)-en-28-oic acid (**T1a**).

3*α*-Metoxy-24-hydroxylup-20(29)-en-28-oic acid (**T1a**): white, amorphous powder (CHCl_3_), R_f_ = 0.36 (hexane-Me_2_CO, 80:20); ^1^H-NMR (CDCl_3_, 400 MHz) *δ* 4.73 (d, *J* = 2.1 Hz, 1H), 4.60 (dd, *J* = 2.3, 1.4 Hz, 1H), 3.66 (s, 4H), 3.52 (dd, *J* = 10.6, 5.1 Hz, 1H), 3.38 (d, *J* = 11.3 Hz, 1H), 3.08 (br s, 1H), 2.99 (ddd, 1H), 2.25–2.16 (m, 1H), 2.17 (br s, 1H), 1.93–1.85 (m, 3H), 1.68 (s, 3H), 1.66–1.54 (m, 3H), 1.54–1.30 (m, 13H), 1.29–1.10 (m, 3H), 1.04 (dd, *J* = 13.0, 4.6 Hz, 1H), 0.99 (s, 3H), 0.92 (s, 3H), 0.85 (s, 3H), 0.67 (s, 3H). ^13^C-NMR (CDCl_3_, 100 MHz) *δ* 176.9, 150.8, 109.7, 71.5, 56.7, 51.4, 50.5, 49.6, 47.2, 43.1, 42.6, 41.0, 40.6, 38.4, 37.2, 37.1, 34.0, 33.1, 32.3, 30.8, 30.8, 29.8, 26.6, 25.5, 21.0, 19.5, 18.2, 17.9, 16.3, 16.1, 15.0; see Figures S1 and S2.

### 3.4. Synthesis of Compound T1b from T1a

Compound **T1b** was obtained by reacting **T1a** (45.1 mg, 0.09 mmol) with SeO_2_ (51.4 mg, 0.46 mmol) in 10 mL of anhydrous ethanol under reflux for 48 h. After completion, the mixture was cooled to room temperature, and the solvent was removed under reduced pressure. The resulting crude product was treated with 20 mL of water and extracted with dichloromethane (3 × 30 mL). The organic phase was dried over anhydrous Na_2_SO_4_ and concentrated. The residue was purified by column chromatography using a 1:1 mixture of hexane and ethyl acetate, affording 23 mg (49.5%) of 3*α*-metoxy-24-hydroxy-30-oxolup-20(29)-en-28-oic acid (**T1b**) [50,51,52].

3*α*-Metoxy-24-hydroxy-30-oxolup-20(29)-en-28-oic acid (**T1b**): white, amorphous powder (CHCl_3_), R_f_ = 0.38 (hexane-EtOAc, 50:50); ^1^H-NMR (CDCl_3_, 400 MHz) *δ* 9.52 (s, 1H), 6.28 (s, 1H), 5.90 (s, 1H), 3.67 (s, 3H), 3.65 (pt, 1H), 3.51 (d, *J* = 11.3 Hz, 1H), 3.38 (d, *J* = 11.3 Hz, 1H), 3.32 (ddd, 1H), 2.97 (br s, 1H), 2.29–2.25 (m, 1H), 2.24 (br s, 1H), 2.13–2.04 (m, 1H), 1.98–1.86 (m, 4H), 1.70–1.56 (m, 3H), 1.54–1.24 (m, 11H), 1.26–1.14 (m, 3H), 0.94 (dd, *J* = 13.0, 4.1 Hz, 1H), 0.96 (s, 3H), 0.90 (s, 3H), 0.84 (s, 3H), 0.67 (s, 3H). ^13^C-NMR (CDCl_3_, 100 MHz) *δ* 194.1, 175.7, 155.6, 133.0, 70.5, 55.8, 50.5, 50.5, 49.9, 49.3, 42.1, 42.1, 41.6, 39.9. 39.6, 37.2, 36.2, 36.0, 33.0, 32.1, 31.3, 31.1, 28.7, 26.4, 25.6, 19.9, 17.2, 16.9, 15.3, 15.0, 13.9; see Figures S3 and S4.

### 3.5. Synthesis of Compounds T1c and T1d from T1

A solution of compound **T1** (50 mg, 0.11 mmol) and SeO_2_ (58.7 mg, 0.53 mmol) in 10 mL of anhydrous ethanol was prepared and subjected to the same procedure described for the synthesis of **T1b**. The resulting residue was purified by column chromatography (hexane-EtOAc, 1:1), yielding 5.4 mg (17%) of 3*α*,24-dihydroxy-30-oxolup-20(29)-en-28-oic acid (**T1c**) and 6.0 mg (19%) of the subproduct 3*α*,24,30-trihydroxylup-20(29)-en-28-oic acid (**T1d**).

3*α*,24-Dihydroxy-30-oxolup-20(29)-en-28-oic acid (**T1c**): white, amorphous powder (MeOH), R_f_ = 0.40 (hexane-EtOAc, 50:50); ^1^H-NMR (CD_3_OD, 400 MHz) *δ* 9.51 (s, 1H), 6.39 (s, 1H), 6.02 (s, 1H), 3.59 (pt, 1H), 3.51 (d, *J* = 10.9 Hz, 1H), 3.39 (ddd, 1H), 3.33 (d, *J* = 10.7 Hz, 1H), 2.38–2.24 (m, 1H), 2.26 (br s, 1H), 2.14–2.02 (m, 1H), 1.98–1.86 (m, 4H), 1.68–1.56 (m, 3H), 1.54–1.30 (m, 13H), 1.26–1.14 (m, 3H), 0.94 (dd, *J* = 12.7, 4.3 Hz, 1H), 1.01 (s, 3H), 0.98 (s, 3H), 0.88 (s, 3H), 0.70 (s, 3H). ^13^C-NMR (CD_3_OD, 100 MHz) *δ* 196.9, 180.6, 158.8, 135.0, 76.5, 71.5, 57.9, 52.4, 51.6, 44.5, 43.6, 41.9, 41.3, 39.5, 38.2, 38.1, 35.1, 34.2, 33.7, 33.3, 30.8, 30.5, 28.7, 26.7, 22.0, 19.0, 17.8, 16.9, 16.7, 15.0; see Figures S5 and S6.

3*α*,24,30-Trihydroxylup-20(29)-en-28-oic acid (**T1d**): white, amorphous powder (MeOH), R_f_ = 0.22 (hexane-EtOAc, 50:50); ^1^H-NMR (CD_3_OD, 400 MHz) *δ* 4.96 (s, 1H), 4.88 (s, 1H), 4.05 (*J*_AB_ = 15.8, 2H), 3.59 (pt, 1H), 3.51 (d, *J* = 11.0 Hz, 1H), 3.32 (d, *J* = 11.0 Hz, 1H), 2.91 (ddd, 1H), 2.38–2.24 (m, 1H), 2.26 (br s, 1H), 2.10–1.99 (m, 1H), 1.93–1.83 (m, 2H), 1.78–1.68 (t, 1H), 1.64–1.24 (m, 18H), 1.22–1.11 (m, 3H), 0.94 (dd, *J* = 12.7, 4.3 Hz, 1H), 1.05 (s, 3H), 0.98 (s, 3H), 0.89 (s, 3H), 0.71 (s, 3H). ^13^C-NMR (CD_3_OD, 100 MHz)*δ*180.7, 156.5, 106.8, 76.5, 71.5, 65.2, 57.7, 51.8, 51.1, 44.5, 44.1, 43.6, 42.0, 41.3, 39.5, 38.2, 38.1, 35.1, 34.3, 33.5, 33.4, 30.9, 28.0, 26.7, 22.1, 19.1, 17.9, 16.9, 16.7, 15.2; see Figures S7 and S8.

### 3.6. Synthesis of Compound T1e from T1

Compound **T1** (20.0 mg, 0.04 mmol) was dissolved in 10 mL of acetic anhydride (Ac_2_O) and 0.5 mL of pyridine (C_5_H_5_N), and the mixture was stirred at room temperature for 72 h. Upon completion, the reaction was quenched with water and extracted by liquid–liquid extraction using H_2_O-EtOAc (1:2 × 2 and 1:1). The organic phase was washed with 5% HCl and brine [53], dried over anhydrous Na_2_SO_4_, filtered, and concentrated under reduced pressure. The crude product was purified by column chromatography (hexane-Me_2_CO, 95:5) to give 12.3 mg (61.5%) of 3*α*-acetyl-24-hydroxylup-20(29)-en-28-oic acid (**T1e**).

3*α*-Acetyl-24-hydroxylup-20(29)-en-28-oic acid (**T1e**): white, amorphous powder (CHCl_3_), R_f_ = 0.46 (hexane-Me_2_CO, 80:20); ^1^H-NMR (CDCl_3_, 400 MHz) *δ* 4.82 (t, *J* = 2.5 Hz, 1H), 4.74 (d, *J* = 2.3 Hz, 1H), 4.60 (s, 1H), 4.02 (d, *J* = 10.4 Hz, 1H), 3.80 (d, *J* = 10.4 Hz, 1H), 3.00 (td, *J* = 10.7, 4.7 Hz, 1H), 2.28 (dt, *J* = 12.7, 3.0 Hz, 1H), 2.19 (td, *J* = 12.3, 3.6 Hz, 1H), 2.02 (s, 3H), 1.97 (d, *J* = 7.4 Hz, 1H). 1.88–1.77 (m, 1H), 1.69 (s, 3H), 1.67–1.60 (m, 2H), 1.58–1.15 (m, 18H), 1.14–1.04 (m, 2H), 1.02 (s, 3H), 0.97 (s, 3H), 0.94 (s, 3H), 0.88 (s, 3H). ^13^C-NMR (CDCl_3_, 100 MHz) *δ* 171.5, 170.5, 150.5, 109.9, 73.2, 70.7, 56.5, 50.5, 49.4, 47.7, 47.0, 42.7, 40.8, 39.7, 38.5, 37.2, 37.2, 33.9, 33.6, 32.3, 30.7, 29.8, 25.6, 22.3, 21.4, 21.0, 20.8, 19.5, 18.7, 17.3, 16.2, 15.0; see Figures S9 and S10.

### 3.7. Leukemia Cell Lines

The acute myeloid leukemia cell line (THP-1, ATCC TIB-202) and the acute lymphoblastic leukemia cell lines (CCRF-CEM, ATCC CCL-19; JURKAT CLONE E6-1, ATCC TIB-152; MOLT-4, ATCC CRL-1582; REH, ATCC CRL-8286) were obtained from the American Type Culture Collection (ATCC) and kindly provided by María Antonieta Chávez González from Unidad de Investigación Médica en Enfermedades Oncológicas, UMAE Hospital de Oncología, Centro Médico Nacional Siglo XXI, IMSS. All experimental procedures were approved by the Ethics and Scientific Committee at IMSS.

Cell lines were cultured in RPMI 1640 medium (Roswell Park Memorial Institute medium) supplemented with 10% fetal bovine serum (FBS) and 1% penicillin–streptomycin. Cultures were maintained at 37 °C in a humidified incubator with 5% CO_2_. All biological evaluations were performed using cell between passages 3 and 4.

### 3.8. Compounds and Controls

All compounds were dissolved in dimethyl sulfoxide (DMSO) at a concentration of 20 mg/mL. In all assays, culture medium containing 0.1% DMSO was used as the negative control, while dasatinib was included as the positive control. Normal mononuclear cells (MNCs) were included as the normal cell control. All experiments were performed in triplicate.

### 3.9. Cell Viability Assay in Leukemic Cell Lines and Normal Mononuclear Cells

Leukemic cell lines and normal mononuclear cells (MNCs) were used to evaluate the cytotoxicity of the compounds. Leukemic cells (1 × 10^5^ cells/well) were cultured in 96-well plates and treated with increasing concentrations of compounds (0.02, 0.2, 2.0, and 20 µM) for 48 h.

Normal mononuclear cells (MNCs) were isolated from peripheral blood mononuclear cells obtained from healthy human donors. Cell collection was conducted in accordance with institutional guidelines, including written informed consent from each participant. All procedures were approved by Ethics and Scientific Committee of the Mexican Institute of Social Security (IMSS, ethical approval number: CNIC R-2021-785-025).

The MNCs were purified using FicollPaque Plus (Pharmacia Biotech, Uppsala, Sweden) by centrifugation at 400× *g* for 30 min at room temperature, following the manufacturer’s protocol [54,55]. The resulting cells were resuspended in RPMI-1640 medium supplemented with 10% fetal bovine serum (FBS) and counted using a hemocytometer after trypan blue staining, confirming a viability of 95% [56]. A total of 1 × 10^5^ cells/well were cultured in 96-well plates and treated with various concentrations of compounds (25, 50, 100, and 200 µM) for 48 h.

After incubation, both leukemic cells and MNCs were collected, washed with phosphate-buffered saline (PBS), and stained with DAPI (500 ng/mL) for 15 min in darkness, following the manufacturer’s instructions [57]. Fluorescence was analyzed using a FACSVerse flow cytometer (BD Bioscience, San Jose, CA, USA).

### 3.10. Apoptosis Assay

Apoptosis induction was evaluated by flow cytometry. Cells (1 × 10^5^ cells/well) were cultured in 96-well plates and exposed to the compounds at two concentrations (½ and 1 × IC_50_). After 48 h of treatment, the cells were washed with phosphate-buffered saline (PBS) and stained with annexin V-FITC (BD Bioscience) for 15 min in the dark. Following a second PBS wash, the cells were stained with DAPI (500 ng/mL) for 15 min in darkness. The stained samples were subsequently analyzed on a FACSVerse cytometer (BD Bioscience, San Jose, CA, USA) [58].

### 3.11. In Silico Studies

Pharmacokinetic and physicochemical properties related to ADME (absorption, distribution, metabolism, and excretion) were predicted for the studied compounds using SwissADME [48]. Parameters analyzed included molecular weight (MW), numbers of hydrogen bond acceptors (nHBA) and donors (nHBD), lipophilicity (cLogP), number of rotatable bonds (nROTB), topological polar surface area (TPSA), and additional pharmacokinetic parameter descriptors.

For molecular docking analysis, three target protein structures were retrieved from the RCSB Protein Data Bank [59]: (1) the anti-apoptotic protein BCL-2 bound to a BH3 domain-specific inhibitor (PDB-ID: 4IEH), (2) the tyrosine kinase domain of epidermal growth factor receptor (EGFR) complexed with erlotinib (PDB-ID: 1M17), and (3) FMS-like tyrosine kinase 3 (FLT3) in complex the inhibitor quizartinib (PDB ID: 4RT7).

Molecular Operating Environment (MOE) software was used to perform molecular docking analysis and visualization [40]. First, MOE’s SiteFinder tool was used to identify the best binding site in the three-dimensional structure of the receptor. This tool evaluates possible binding sites and provides a ranking of them, allowing the selection of the area of highest affinity for the ligand.

Once the binding site was identified, molecular docking was performed using the Triangle Matcher, an MOE docking algorithm that specializes in the search and evaluation of ligand docking positions at the receptor binding site, considering geometric and steric interactions. The London dG scoring method was used both for the calculation of the energy during ligand docking at the binding site and in the refinement of conformations. During the docking process, a total of 300 ligand conformations were generated at the selected binding site. To improve the quality of the obtained poses and reduce the computational complexity, a set of 25 conformations showing the best energy scores was refined [40].

### 3.12. Analysis of Results

Flow cytometry data were analyzed using FlowJo^TM^ software (version 10.9). Cell viability result IC_50_ values—defined as the compound concentrations that inhibit 50% of cell proliferation—were determined from dose–response curves using GraphPad Prism software (version 9). All experiments were performed in triplicate, and results were expressed as means ± standard errors of the means (SEMs). Statical analysis was performed using one-way analysis of variance (ANOVA), followed by Dunnett’s post hoc test to compare treated groups against the control. A *p*-value < 0.05 was considered statistically significant.

## 4. Conclusions

The triterpene 3*α*,24-dihydroxylup-20(29)-en-28-oic acid (**T1**), isolated from *Phoradendron wattii*, was modified and characterized by ^1^H- and ^13^C-NMR experiments. From **T1**, five derivatives were generated; among them, 3*α*,24-dihydroxy-30-oxolup-20(29)-en-28-oic acid (**T1c**) induced inhibition of cell proliferation and cell death by apoptosis in ALL and AML cell lines. The **T1c** derivative is a promising candidate because it exhibited minimal effects on MNC cells. Molecular docking analysis showed that **T1c** has a higher affinity for EGFR protein kinase than its precursor molecule (**T1**). These findings suggest that the introduction of an *α*,*β*-unsaturated aldehyde group at C-30 in pentacyclic triterpenes significantly enhances the biological activity on leukemia cell lines, highlighting the potential of **T1c** in future research for leukemia treatment.

## Figures and Tables

**Table 1:** The mean inhibitory concentration of triterpenes and derivatives evaluated against leukemia cell lines.

Compound	IC_50_ µM (SI)				
	CCRF-CEM	REH	JURKAT	MOLT-4	THP-1
**T1**	N/A	N/A	N/A	N/A	N/A
**T1a**	N/A	64.22 ± 4.7	N/A	N/A	N/A
**T1b**	6.87 ± 0.6 (1.59)	9.46 ± 3.2 (1.13)	14.97 ± 1.6 (0.72)	4.05 ± 0.1 (2.66)	11.04 ± 0.1 (0.97)
**T1c**	17.79 ± 1.0 (75.02)	24.92 ± 0.4 (53.55)	18.67 ± 1.4 (71.46)	13.86 ± 0.7 (96.30)	12.90 ± 0.1 (103.46)
**T1d**	N/A	N/A	N/A	N/A	N/A
**T1e**	N/A	N/A	N/A	N/A	N/A
**T2**	27.36 ± 0.9 (19.48)	46.74 ± 1.6 (11.40)	62.27 ± 2.3 (8.56)	N/A	N/A
Dasatinib	10.76 ± 1.2	5.43 ± 0.3	5.26 ± 0.5	2.63 ± 0.1	4.66 ± 0.3

SI: selectivity index (ratio of IC_50_ in the MNC line over IC_50_ in the leukemia cell line); N/A: not active.

**Table 2:** The mean inhibitory concentrations of triterpenes and derivatives evaluated against peripheral blood mononuclear cells.

IC_50_ µM			
Compound	MNCs	Compound	MNCs
**T1**	275.01 ± 3.1	**T1d**	N/A
**T1a**	N/A	**T1e**	N/A
**T1b**	10.78 ± 1.3	**T2**	532.98 ± 6.1
**T1c**	1334.73 ± 4.3	Dasatinib	N/A

MNCs: peripheral blood mononuclear cells; N/A: not active; ≤200 µM.

**Table 3:** Docking score for compounds **T1**, **T1c**, **T2**, and betulinic acid.

Compound	Score (kcal/mol)		
	BCL-2	EGFR (TK Domain)	FLT3
**T1**	−10.12	−12.75	−14.05
**T1c**	−10.23	−14.50	−14.07
**T2**	−11.59	−15.00	−14.03
Betulinic acid	−9.74	−12.73	−13.64
Co-crystalized inhibitor	−17.72	−13.97	−18.17

**Table 4:** In silico predicted physicochemical and pharmacokinetic parameters of **T1**, **T1c**, **T2**, and betulinic acid.

		Compounds			
		T1	T1c	T2	BA
Molecular Weight (g/mol)		472.70	486.68	486.68	456.70
Physicochemical Parameters	nHBA	4	5	5	3
	nHBD	3	3	3	2
	cLogP	5.52	4.66	4.69	6.14
	nROTB	3	4	4	2
	TPSA (Å^2^)	77.76	94.83	94.83	57.53
Pharmacokinetic Parameters	GI absorption	High	High	High	Low
	CYP1A2 inhibitor	No	No	No	No
	CYP2C19 inhibitor	No	No	No	No
	CYP2C9 inhibitor	Yes	No	No	Yes
	CYP2D6 inhibitor	No	No	No	No
	CYP3A4 inhibitor	No	No	No	No

nHBA: num. of H-bond acceptors; nHBD: num. of H-bond donors; cLogP: lipophilicity (consensus Log Po/w); nROTB: num. of rotatable bonds; TPSA: topological polar surface area; GI absorption: gastrointestinal absorption; CYP1A2: cytochrome P450 1A2; CYP2C19: cytochrome P450 2C19; CYP2C9: cytochrome P450 2C9; CYP2D6: cytochrome P450 2D6; CYP3A4: cytochrome P450 3A4; BA: betulinic acid.

## Data Availability

Data are contained within the article and Supplementary Materials.

## Supplementary Materials

The following supporting information can be downloaded at: https://www.mdpi.com/article/10.3390/molecules30153193/s1. **Figures S1 and S2**. Nuclear magnetic resonance spectra of compound **T1a** [**Figure S1**. ^1^H-NMR (CDCl_3_, 400 MHz), **Figure S2**. ^13^C-NMR (CDCl_3_, 100 MHz)]. **Figures S3 and S4**. Nuclear magnetic resonance spectra of compound **T1b** [**Figure S3**. ^1^H-NMR (CDCl_3_, 400 MHz), **Figure S4**. ^13^C-NMR (CDCl_3_, 100 MHz)]. **Figures S5 and S6**. Nuclear magnetic resonance spectra of compound **T1c** [**Figure S5**. ^1^H-NMR (CD_3_OD, 400 MHz), **Figure S6.** ^13^C-NMR (CD_3_OD, 100 MHz)]. **Figures S7 and S8**. Nuclear magnetic resonance spectra of compound **T1d** [**Figure S7**. ^1^H-NMR (CD_3_OD, 400 MHz), **Figure S8**. ^13^C-NMR (CD_3_OD, 100 MHz)]. **Figures S9 and S10**. Nuclear magnetic resonance spectra of compound **T1e** [**Figure S9**. ^1^H-NMR (CDCl_3_, 400 MHz), **Figure S10**.^13^C-NMR (CDCl_3_, 100 MHz)].
