# Supplementary material for: Semi-Synthesis, Anti-Leukemia Activity, and Docking Study of Derivatives from 3α,24-Dihydroxylup-20(29)-en-28-Oic Acid
Source: Molecules. 2025 Jul 30;30(15):3193. doi: 10.3390/molecules30153193 (PMC12348585; doi:10.3390/molecules30153193)
Supplement: Supplementary file 1 [file molecules-30-03193-s001.zip › molecules-3746304-supplementary.pdf]

Supplementary Material

Mario J. Noh-Burgos [1,2], Sergio García-Sánchez [3], Fernando J. Tun-Rosado [4], Antonieta Chávez-González [3], Sergio R. Peraza-Sánchez [1], Rosa E. Moo-Puc [5]

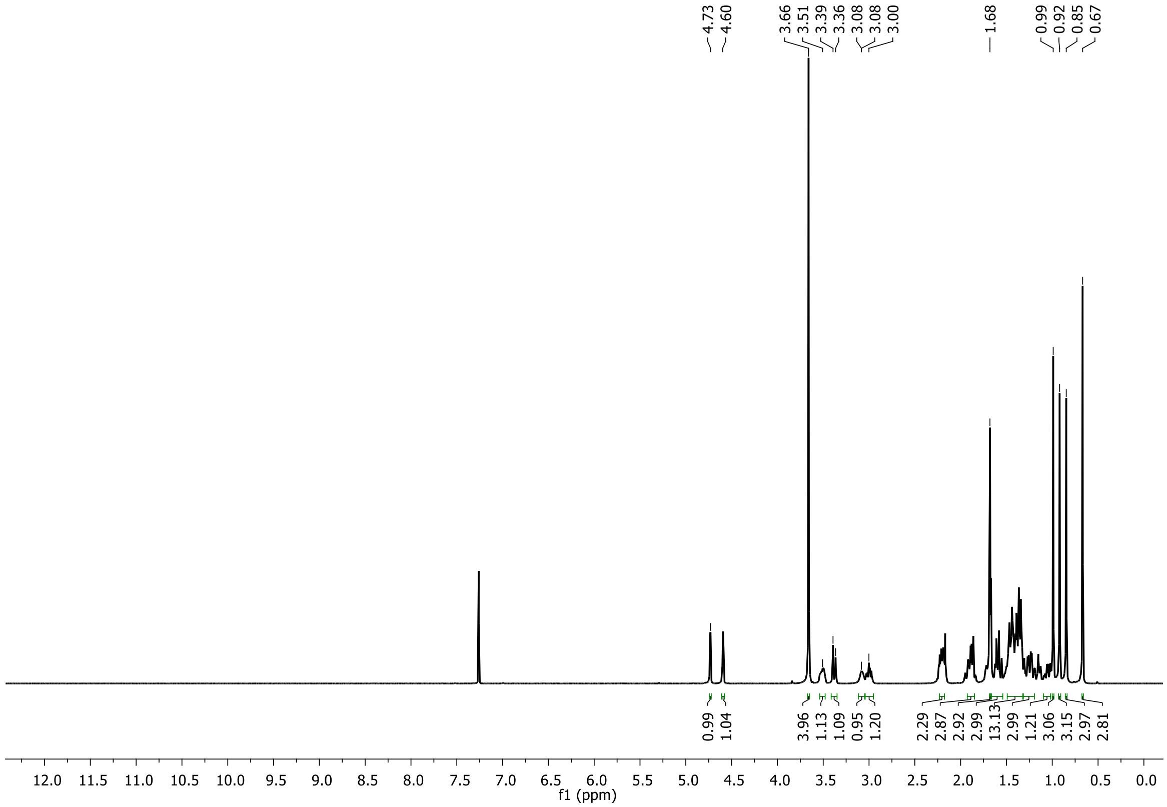

Figure S1. ^{1}H-NMR ($CDCl_3$, 400 MHz) spectrum of 3α-metoxy-24-hydroxylup-20(29)-en-28-oic acid (**T1a**)

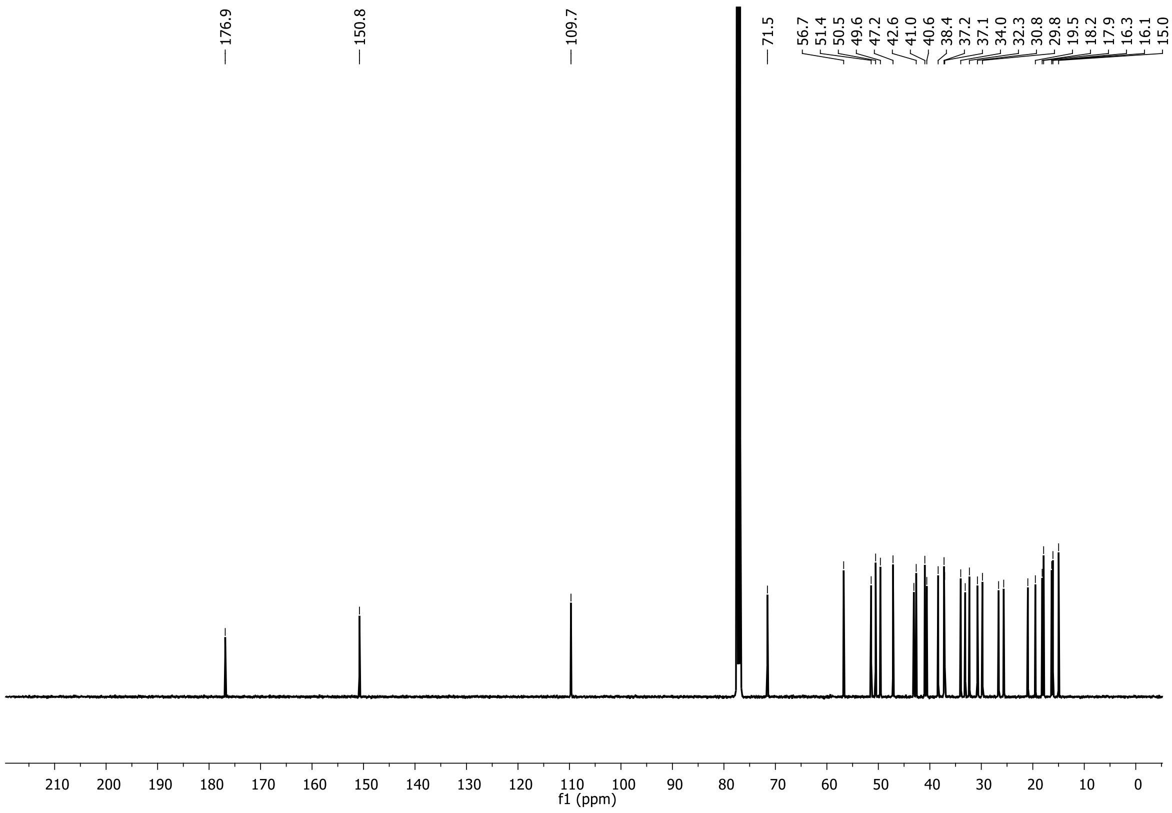

Figure S2. ^{13}C-NMR ($CDCl_3$, 100 MHz) spectrum of 3α-metoxy-24-hydroxylup-20(29)-en-28-oic acid (**T1a**).

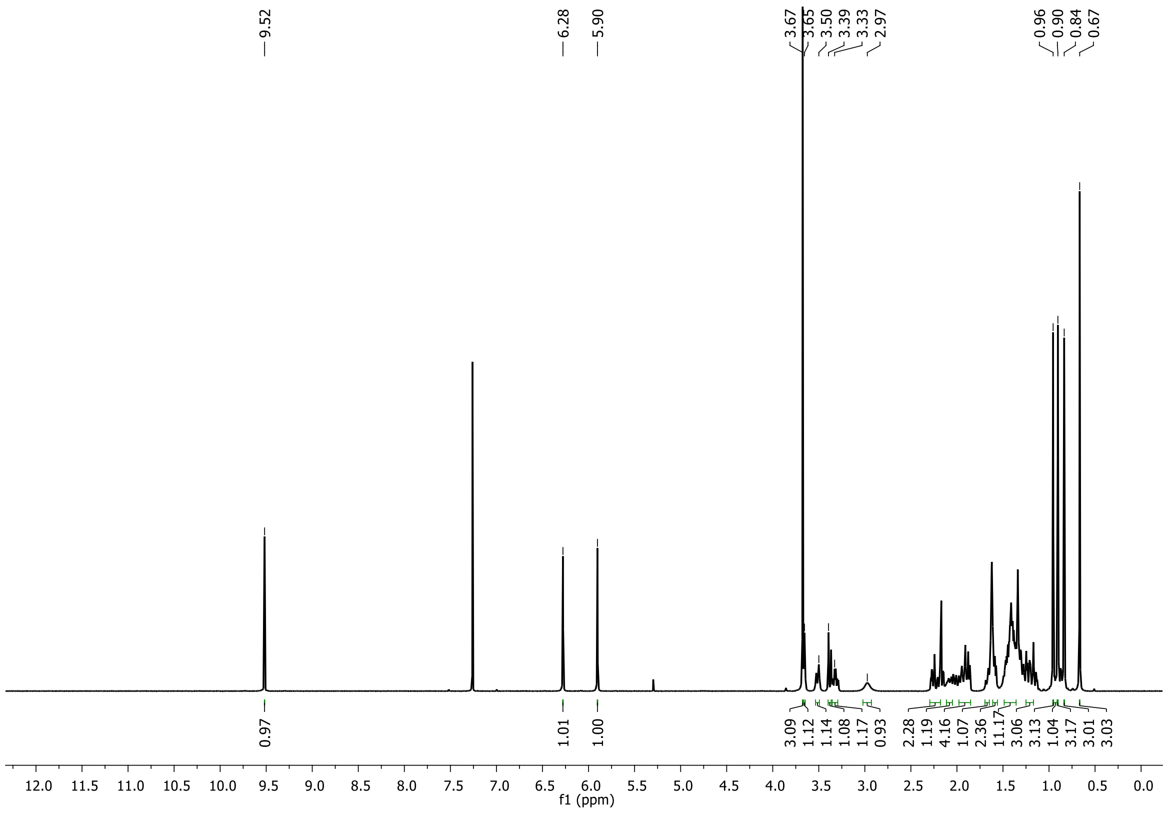

Figure S3. ^{1}H-NMR ($CDCl_3$, 400 MHz) spectrum of 3α-metoxy-24-hydroxy-30-oxolup-20(29)-en-28-oic acid (**T1b**).

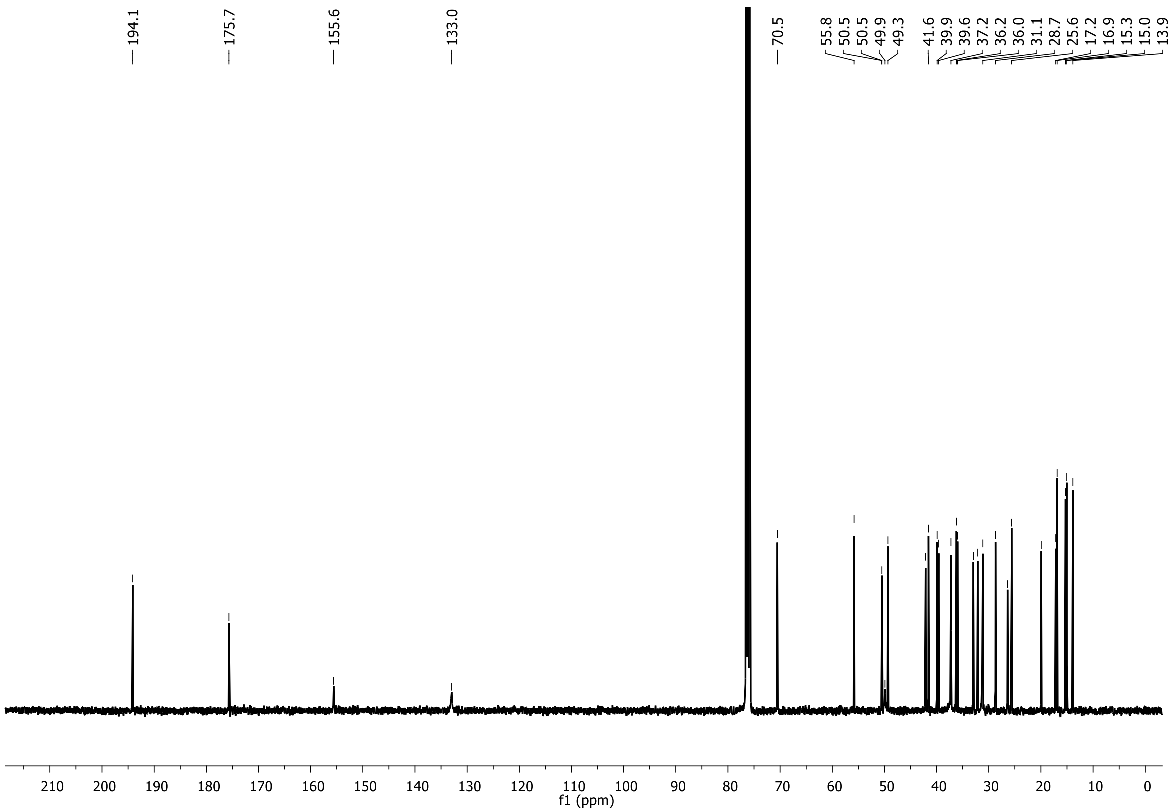

Figure S4. ^{13}C-NMR ($CDCl_3$, 100 MHz) spectrum of 3α-metoxy-24-hydroxy-30-oxolup-20(29)-en-28-oic acid (**T1b**).

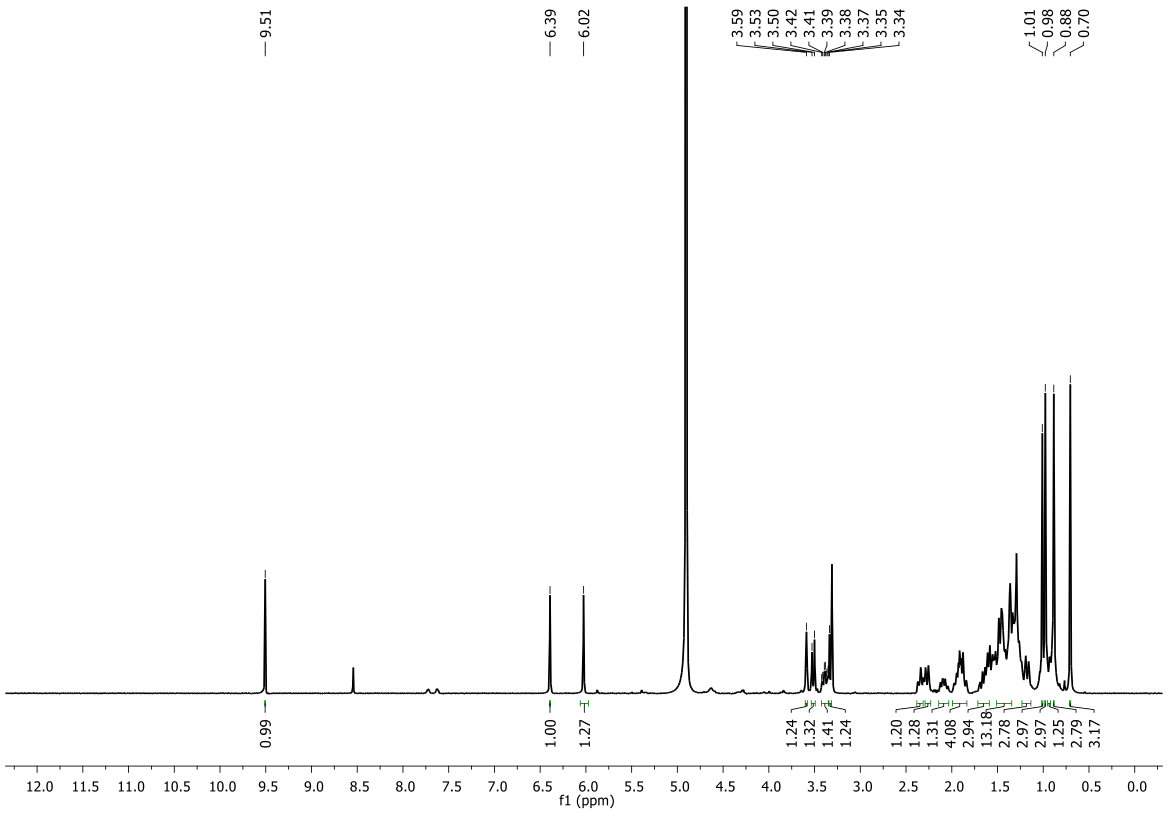

Figure S5. ^{1}H-NMR (CD_3OD, 400 MHz) spectrum of 3α,24-hydroxy-30-oxolup-20(29)-en-28-oic acid (**T1c**).

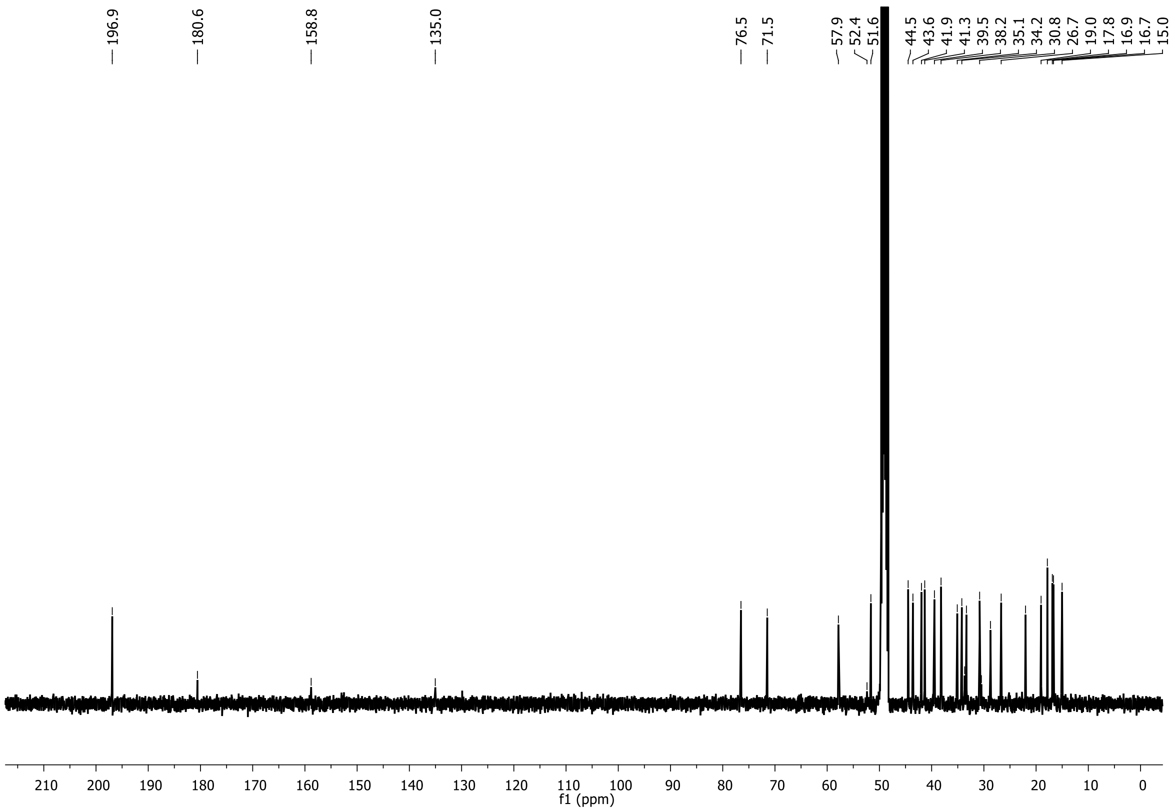

Figure S6. ^{13}C-NMR (CD_3OD, 100 MHz) spectrum of 3α,24-hydroxy-30-oxolup-20(29)-en-28-oic acid (**T1c**).

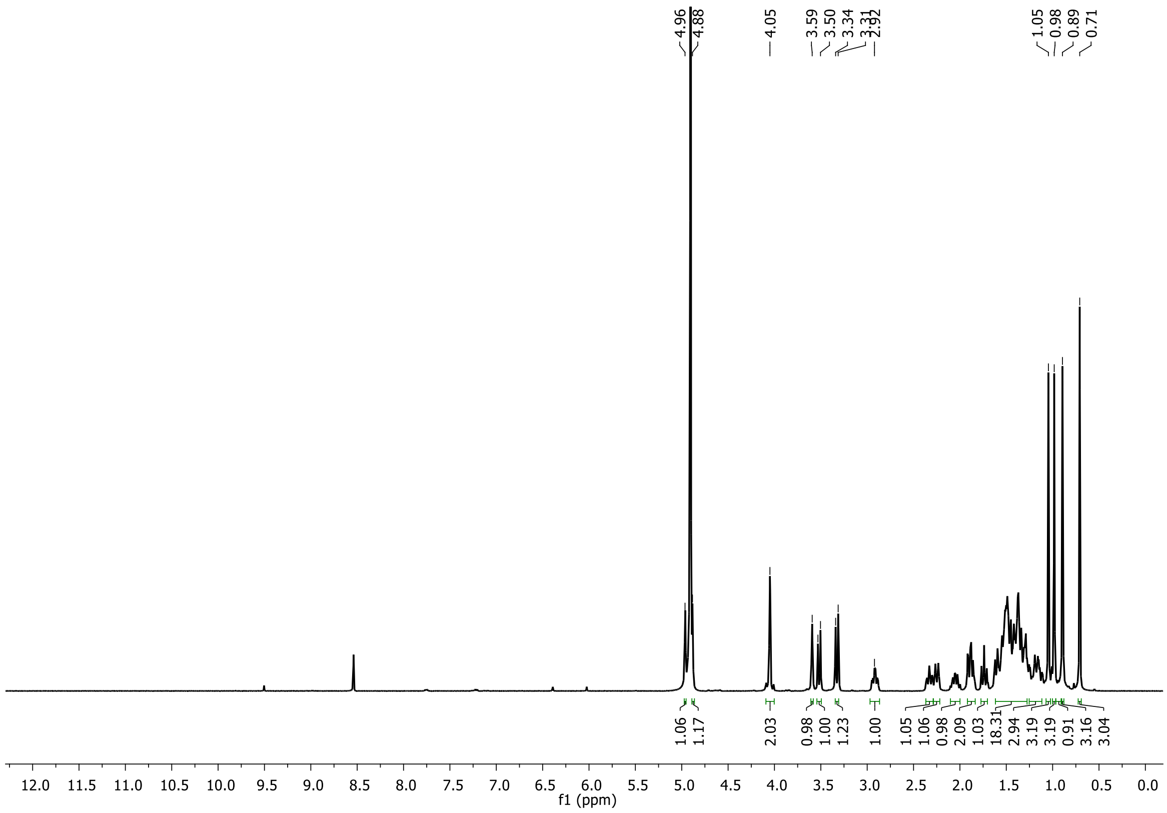

Figure S7. ¹H-NMR (CD_3OD, 400 MHz) spectrum of 3α,24,30-trihydroxylup-20(29)-en-28-oic acid (**T1d**).

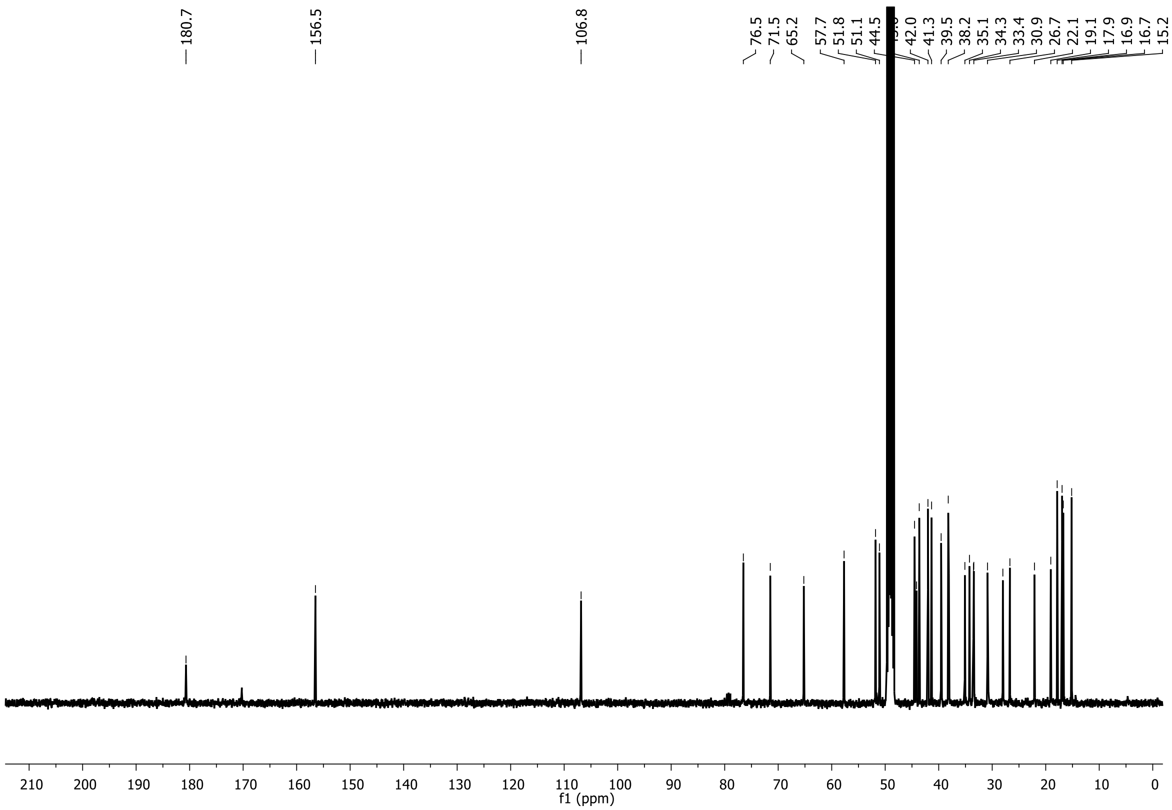

Figure S8. ^{13}C-NMR (CD_3OD, 100 MHz) spectrum of 3α,24,30-trihydroxylup-20(29)-en-28-oic acid (**T1d**).

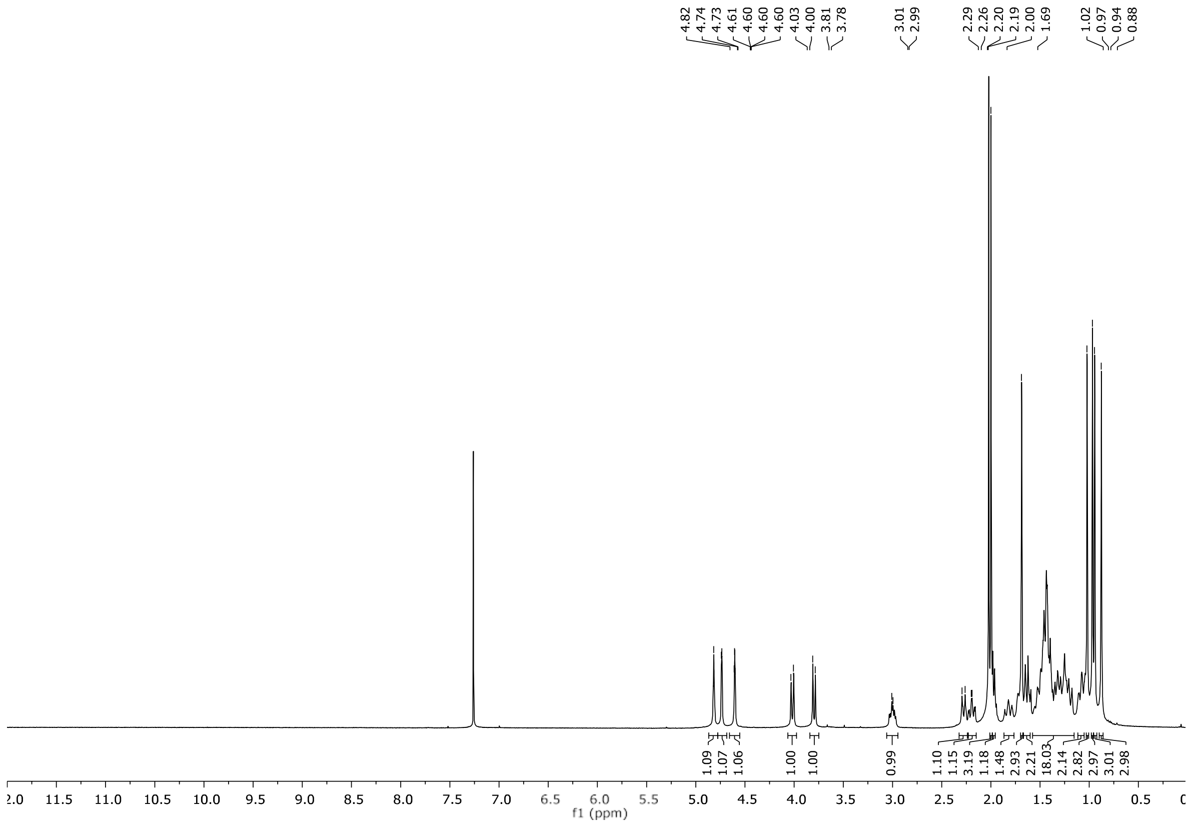

Figure S9. ^{1}H-NMR ($CDCl_3$, 400 MHz) spectrum of 3α-acetyl-24-hydroxylup-20(29)-en-28-oic acid (**T1e**).

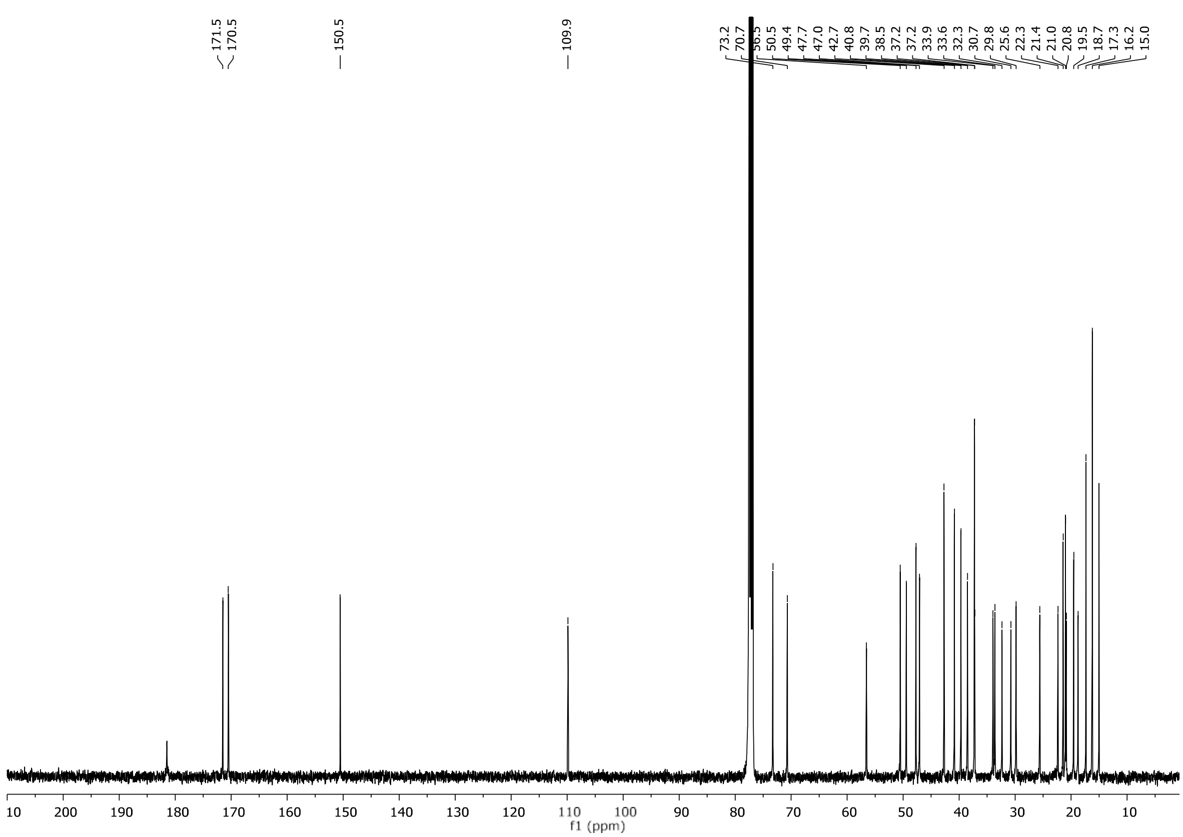

Figure S10. ^{13}C-NMR ($CDCl_3$, 100 MHz) spectrum of 3α-acetyl-24-hydroxylup-20(29)-en-28-oic acid (**T1e**).
